# Combined versus single treatment regimens for keloid therapy using serial intralesional corticosteroid injections, surgical excision, silicone- and/or cryotherapy

**DOI:** 10.1016/j.jpra.2021.05.011

**Published:** 2021-06-03

**Authors:** C. Jacobs, J. Wilmink

**Affiliations:** a Department of Plastic and Reconstructive Surgery, Máxima Medisch Centrum Veldhoven, The Netherlands; b Maastricht University Faculty of Health, Medicine and Life Sciences

**Keywords:** Keloid, Combination therapy, Monotherapy, Cryotherapy, Protocolled corticosteroid injections

## Abstract

Efficacy comparison of several regimens in treating keloids as combined or standalone therapies could provide essential information for selecting appropriate therapy. This study retrospectively evaluated the treatment efficacy of corticosteroid injections, excision, silicone, cryotherapy, or combinations of these for treating keloids. Additionally, the use of corticosteroid injection schemes and combined cryotherapy regimens were analysed.

Retrospective chart analysis was performed on 204 keloids treated patients at the plastic surgery department of the Máxima Medical Centre between 2009 and 2018. The patient's age, gender, treatment, anatomic location, scar aetiology, previous therapy, scar recurrence, additional therapy, and follow-up duration were retrieved. Treatment efficacy was assessed through treatment failure, defined by the recurrence or lack of response. Kaplan–Meier and Cox survival analyses were performed to compare treatment efficacy between the different regimens.

Monotherapies exhibited a significantly higher chance of treatment failure (HR 2.4, 95% CI 1.4–4.2, p<0.05) when compared to combined therapies.

Sporadic corticosteroid injections demonstrated more treatment failure overall (HR 3.5 95% CI 1,6–7,3; p=0.001), but did not differ significantly from injection schemes.

Combined cryotherapy efficacy did not differ significantly from the other combined regimens (HR 1,6 95% CI 0,5–5,1; p=0.401).

Combined therapies exhibited clear superiority over monotherapies. Sporadic corticosteroid injections demonstrated inferior results compared to all other therapies. Combined cryotherapy cases were insufficient, and more data are required for proper assessment. Future prospective assessments of corticosteroid injection schemes and combined regimens are warranted.

## Introduction

Abnormal scar formation following cutaneous injury in predisposed circumstances can lead to keloid or hypertrophic scarring.^1^, ^2^, ^3^, ^4^ Unlike hypertrophic scars, keloid tissue spreads beyond the borders of the enticing wound and does not exhibit spontaneous regression.^2^^,^^3^^,^^5^ Besides being a therapeutic challenge for physicians, keloids also potentiate psychological and functional impairments in the affected patients.^1^^,^^4^

Multiple therapeutic modalities and possible combinations of therapies have been described for treating keloids; however, the most optimal intervention or combination of regimens has yet to be found and a standard regimen remains to be established.^1^^,^^3^^,^^5^, ^6^, ^7^ Treatment efficacy reported for different regimens also varies greatly in available literature and recommendations are not consistent.^1^^,^^8^^,^^9^ Hence, high recurrence rates occur after therapeutic intervention, and physicians lack effective protocols or established treatment regimens that can be adhered to.^1^^,^^2^^,^^6^^,^^7^

Possible interventions for keloids frequently described in the literature include cryotherapy, intralesional corticosteroid injections, silicone therapy, surgical excision, or combinations of these regimens.^1^, ^2^, ^3^, ^4^^,^^6^^,^^7^ The use of corticosteroid injection schemes,^1^^,^^10^^,^^11^ and cryotherapy as an adjunct to excision or corticosteroids have also been described, though it is yet to be confirmed whether these techniques are superior to the conventional approaches.^12^^,^^13^

So far, no studies have compared different combined interventions to different forms of single interventions.^1^^,^^3^^,^^5^^,^^6^^,^^14^ Hence, it remains unclear whether the general use of combined interventions is superior to applying one single intervention. In our centre, keloids have been treated using the aforementioned interventions either as a standalone intervention or as combinations. We have also treated cases using corticosteroid injection schemes or through adjunct cryotherapy.

This study aimed to determine the following:(1) whether there was a superior efficacy in the treatment of keloids when using combined interventions compared to single interventions, (2) whether there was superior efficacy when using corticosteroid injection schemes compared to sporadic injections, and (3) whether the use of adjunctive cryotherapy was superior to other combined regimens.

## Materials and Methods

### Study Design

We conducted a retrospective chart review of patients treated for keloids at the department of Plastic Surgery in the Máxima Medical Centre (MMC) in the Netherlands between 2009 and 2018. The study was approved by the hospital's institutional Medical Research Ethics Committee (METC number N20.018).

We included all keloid treated cases; either by corticosteroid injections, excision, silicone, cryotherapy, or by any of their combinations. Each case was defined as one anatomic location affected by a keloid. Thus, multiple cases could be yielded from one patient who presented keloids at different anatomical locations.

Data collection included patient age, gender, type of treatment, anatomic location of keloid, scar aetiology, previous therapy, recurrence during follow-up, additional therapy, and follow-up duration. Patients who did not turn up for their final outpatient appointments were classified as lost to follow-up.

Unfortunately, skin type/ethnicity could not be determined through this retrospective analysis. The occurrence of adverse effects was not consistently reported, and therefore, could not be incorporated into the analysis.

Therapies were classified as either monotherapy or combined therapy. Monotherapy was defined as standalone cryotherapy, excision, silicone, protocolled corticosteroid injections, or sporadic corticosteroid injections. Corticosteroid injections were subdivided into either protocolled injection schemes or sporadic injections. Combined therapy was defined as any predetermined combination of these regimens and was further subdivided into those with and without cryotherapy.

Efficacy was assessed by the incidence of either recurrence or persistence of keloid during a follow-up period of 400 days, as the average follow-up was 430 days. Treatment was labelled as failed in case of recurrence or persistence during this follow-up.

All analyses were performed using an IBM SPSS Statistics Data Editor (IMB statistics for Windows version 25.0; SPSS, IMB Corp). We applied Kaplan–Meier survival analysis in which treatment efficacy between the different therapies was compared and survival plots representing therapy failure were generated. Statistical significance was assessed with a log-rank test and determined by p-values < .05.

Additionally, Cox regressions were performed to correct for confounders (age, gender, aetiology, previous therapy, and anatomic location of keloid). Hazard ratios (HR) and 95% CI between different therapies were estimated through a Cox proportional hazards model.

## Results

A total of 186 patients yielded 204 keloid cases (110 female and 94 male) with a mean age of 28 (SD±15 years, age range 3–80 years) were treated for keloids between 2009 and 2018. Mean follow-up was 430 days. Mean times until treatment failure per therapy group are given in Table 1. Follow-up visits had intervals ranging from one week to three months. Patient characteristics and distribution among different therapeutic regimens are presented in Table 2.

**Table 1:** Mean Survival Times per Therapy.

Therapy	Mean Survival Time	95% CI
Excision	260	184-335
Cryotherapy	285	219-352
Sporadic corticosteroids	230	176-284
Protocolled corticosteroids	258	195-321
Silicone therapy	245	161-329
Combined regimens with cryotherapy	327	294-360
Combined regimens without Cryotherapy	297	224-370

**Table 2:** 

Patient Characteristics			%
Total cases		204	
Gender	Female	110	53,9
	Male	94	46,1
Mean Age (range) in years	28 (3-80)		
Mean follow up in days (range)	430 (7-2475)		
Anatomic location	Abdomen/Back	23	11,3
	Breast/Areola	11	5,4
	Ear	43	21,1
	Earlobe	31	15,2
	Head/Neck/Face	27	13,2
	Shoulder/Scapula	26	12,7
	Sternum	31	15,2
	Other	12	5,9
Etiology	Unknown	38	18,6
	Acne/Skin condition	18	8,8
	Piercing	22	10,8
	Surgical/Medical Procedure	98	48
	Trauma/Burn/Infection	28	13,7
Previous Therapy	No	151	74,0
	Yes	53	26,0
Mono or Combined Therapy	Monotherapy	121	59,3
	Combined Therapy	83	40,7
Mono or combined Therapy after Therapy Switch	Monotherapy	114	55,9
	Combined Therapy	90	44,1
Therapy groups	Excision	25	12,3
	Cryotherapy	19	9,3
	Sporadic corticosteroid injections	41	20,1
	Serial corticosteroid injections	25	12,3
	Silicone	11	5,4
	Combined *without* cryotherapy	71	34,8
	Combined *with* cryotherapy	12	5,9

Treatment regimens were chosen based on the patient's and physician's shared decision making. Factors influencing treatment choice were anatomic location, previous treatment, and aetiology. Table 3 summarizes the applied regimens and corresponding recurrence or persistence rates.

**Table 3:** Applied therapies with corresponding recurrence/persistence rates.

Therapies	No Recurrence (%)	Recurrence (%)	Persistence (%)	Total
Monotherapy	66 (54,5%)	28 (23,1%)	27 (22,3%)	121
Combined therapy	52 (62,7%)	23 (27,7%)	8 (9,6%)	83
Excision	16 (64%)	8 (32%)	1 (4%)	25
Cryotherapy	11 (57,9%)	5 (26,3%)	3 (15,8%)	19
Sporadic Corticosteroids	22 (53,7%)	5 (12,2%)	14 (34,1%)	41
Protocolled Corticosteroids	11 (44%)	10 (40%)	4 (16%)	25
Silicone	6 (54,5%)	0 (0%)	5 (45,5%)	11
Combined without cryotherapy	47 (66,2%)	18 (25,4%)	6 (8,5%)	71
Combined with Cryotherapy	5 (41,7%)	5 (41,2%)	2 (16,7%)	12
Total	118 (57,8%)	51 (25,0%)	35 (17,2%)	204

### Monotherapy versus Combined Therapy

**Figure 1:**
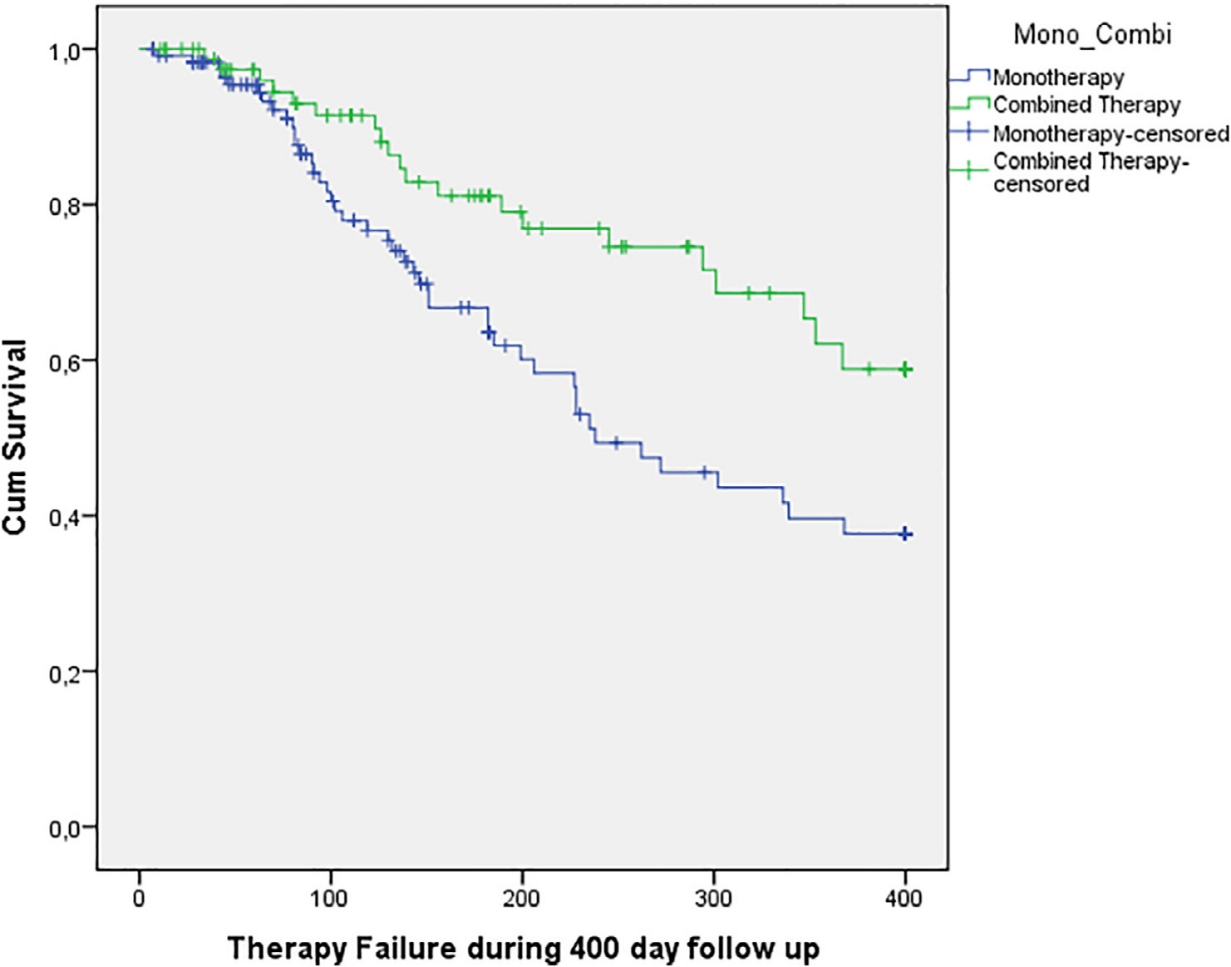
Survival curve reflecting treatment failure during follow-up for monotherapy and combined therapy. There is a clear distinction in efficacy over time favoring combined therapy.

Kaplan–Meier survival analysis revealed statistically significant less treatment failure for combined therapy compared to monotherapy (X^2^(1) = 6.959, p=.008). Twenty combined therapy cases (24%) experienced treatment failure versus 44/121 (36%) monotherapy cases during the follow-up period (Figure 1).

Cox regression analysis also demonstrated a significantly increased hazard for treatment failure in the monotherapy group both with and without adjustment for confounders (Table 4).

**Table 4:** Hazard Ratio for monotherapy compared to combined therapy.

Monotherapy versus combined therapy	HR	95% CI	P
Not adjusted for confounders	2.0	1,2 to 3,4	.01
Adjusted for confounders	2.4	1.4 to 4.2	.002

**Figure 2:**
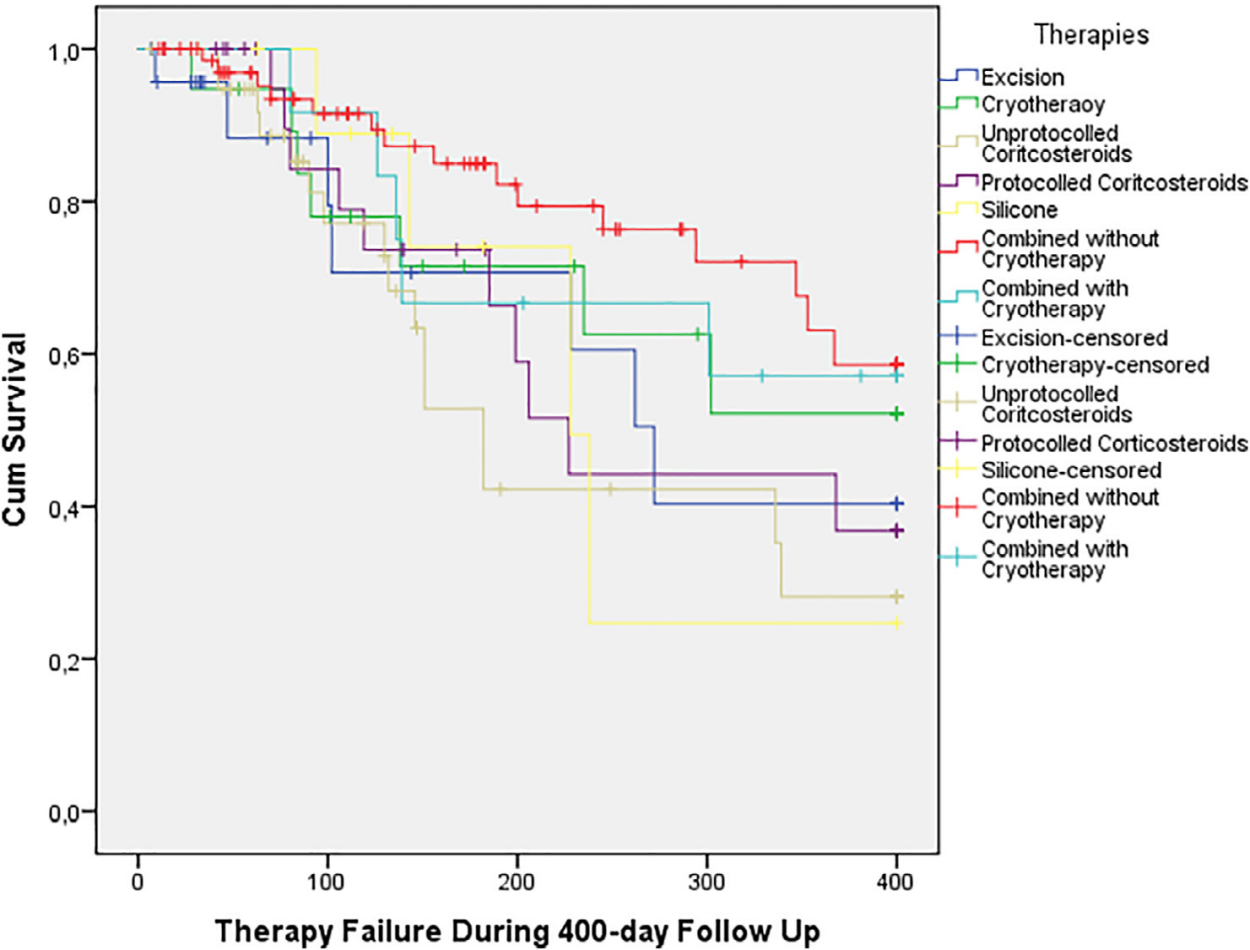
Kaplan– Meier survival functions for separate therapies. The Y-axis “Cum Survival” reflects the fraction of cases not exhibiting recurrence/persistence. No clear pattern between treatment efficacy can be appreciated here/

When assessing all separate regimens, Kaplan–Meier analysis did not exhibit any clear patterns (Figure 2). Furthermore, this analysis was not statistically significant (log-rank X^2^(6) = 9.596, p=0.143).

**Figure 3:**
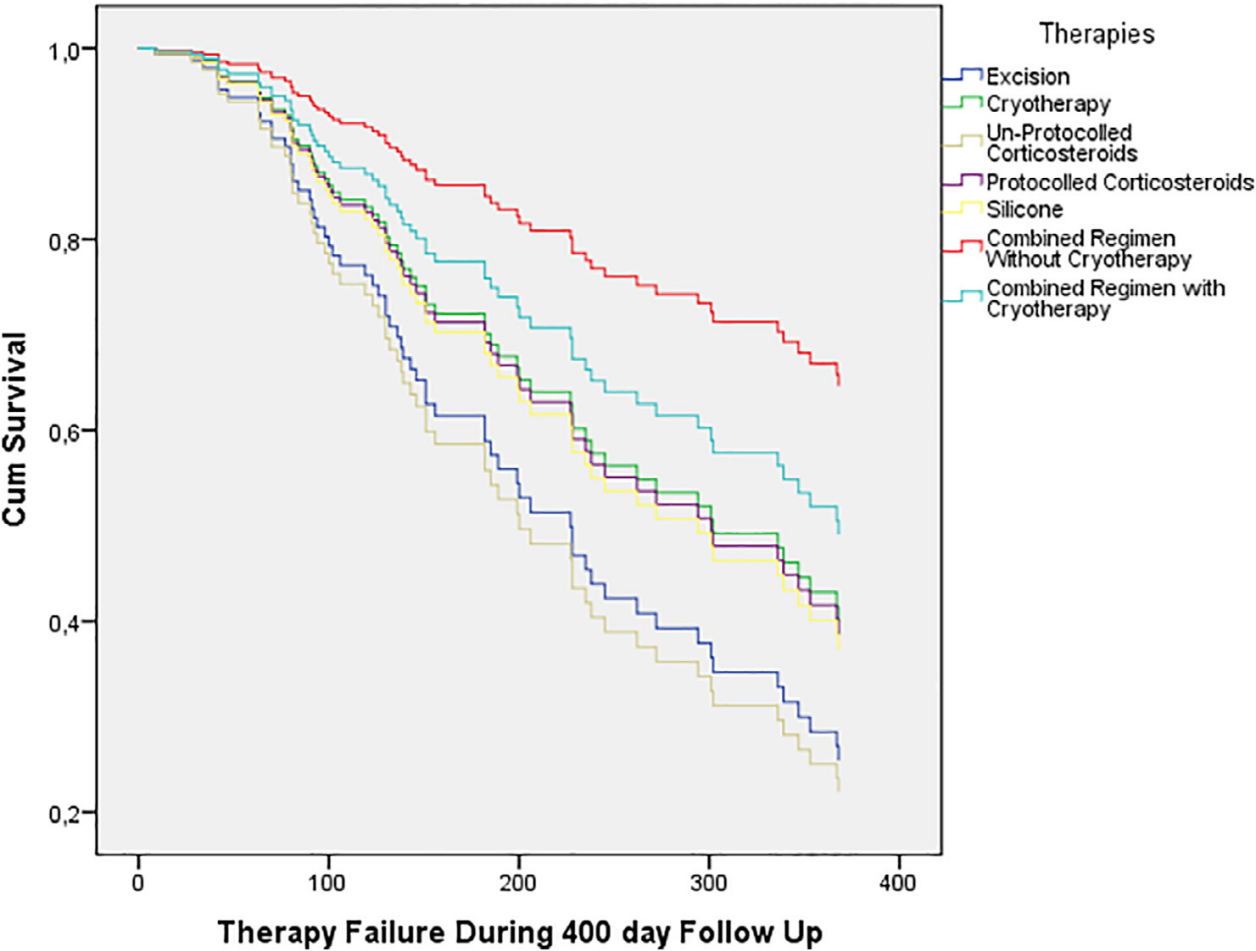
Cox survival curves for separate therapies showing efficacy over time.

Because of the graphs crossing, the proportional hazards assumption hypothesis was tested and this was not rejected. Figure 3 demonstrates the survival curves generated from the Cox regression analysis. The corresponding hazard ratios are given in Table 5*.* Combined regimens without cryotherapy exhibited the highest efficacy, whereas excision and sporadic corticosteroid injections demonstrated more significant recurrence/persistence.

**Table 5:** Adjusted Hazard Ratios for treatment failure. Reference group = combined regimens without cryotherapy.

Regimen	HR	95% CI	P
Excision	3.1	1.2 – 8.5	.024
Cryotherapy	2.1	0.7 – 5.98	.163
Sporadic corticosteroid injections	3.5	1.6 – 7.3	.001
Protocolled corticosteroid injections	2.2	0.9 – 5.3	.084
Silicone therapy	2.3	0.7 – 7.5	.175
Combined regimens with cryotherapy	1.6	0.5 – 5.1	.401
Combined regimens without cryotherapy	1.0	-	-

### Protocolled versus Sporadic Corticosteroid Injections

The Kaplan–Meier Analysis revealed lower failure rates among the protocolled injections group compared to sporadic injections (Figure 2). However, this was not statistically significant (HR 0.6, 95% CI 0.3–1.5, p = 0.307). Meantime until recurrence/persistence was also shorter for sporadic injections (Table 1).

Repeated Cox regression directly comparing protocolled sporadic injections did not exhibit a statistically significant difference in efficacy between these two regimens (adjusted HR 0.6; 95% CI 0.3–1.5; p=.307).

### Combined treatment: with versus without cryotherapy

Only a small number of cases were treated by our injection-excision-cryotherapy scheme. Therefore, we divided combined regimens into those with and without cryotherapy to assess the role of cryotherapy in a combined regimen. All combined regimens, both with and without cryotherapy, exhibited better efficacy than monotherapies. Furthermore, combined regimens without cryotherapy demonstrated the best overall efficacy (Figure 2).

Efficacy between combined regimens with and without cryotherapy was not significantly different; Log-rank: X^2^(6) = 9.596 (p=.143), unadjusted HR: 1,3 (95% CI 0.5–3.7; p=0.583); and adjusted HR: 1,6 (95% CI 0.5–5.1; p=0,401).

## Discussion

### Mono- versus Combined-therapy

The primary aim of this study was to compare the efficacy of combined regimens to monotherapies in the treatment of keloids. Most studies in which combined regimens are compared to single therapies involve only specific comparisons between two or three types of treatments and often have small study populations.^3^^,^^14^, ^15^, ^16^ Therefore, there is a lack of general comparisons between combined and single therapies, and yet, there is a consensus whether combined therapy as a whole is superior to monotherapy when treating keloids.

Our results revealed significant superiority for combined regimens compared to monotherapies. Out of the monotherapies, surgical excision and sporadic corticosteroids exhibited significantly more treatment failure.

Surgical excision was a popular method for treating keloids as it provides immediate scar volume reduction.^3^^,^^16^ However, high recurrence rates reported (45–100%) have led to excision as a standalone treatment, which is being discouraged.^9^ Instead, excision is advised in combination with other treatments.^1^^,^^8^ Its use with complementary therapies has indeed revealed better efficacy.^1^^,^^2^^,^^9^ So far, only one study has analysed the use of different adjunct therapies to excision, in which results were unfortunately inconclusive, owing to small sample size.^3^ The significantly increased hazard for failure as revealed in our results further strengthens the recommendations that excision should not be applied as monotherapy for keloids.

### Protocolled versus sporadic corticosteroid injection schemes

Corticosteroid injections are described as an effective treatment choice for keloids and often advised as first-line therapy for keloids; reported success and recurrence rates, however, vary.^1^^,^^5^^,^^6^^,^^8^^,^^10^ Most reported rates for corticosteroids as a monotherapy are 9–50%.^6^^,^^9^ Numerous intervals for corticosteroid injections have been described;^1^^,^^10^^,^^11^ however, their efficacy has yet to be compared with sporadic injections.

The experience in our clinic is that injection schemes yield superior results compared to sporadic injections. However, our analysis did not present a statistically significant difference between these two methods. As mentioned, sporadic corticosteroid injections were significantly less efficacious than the most effective regimen. The injection schemes, however, were not significantly less efficacious – which implies they may be superior to sporadic injections. Further research comparing corticosteroid injection schemes to sporadic injections is warranted.

Patient subjectivity is an important factor to be considered. The shorter intervals between outpatient visits and more frequent scar assessments may have possibly contributed to the earlier detection of recurrence, whereas patients receiving sporadic injections were assessed less frequently so that recurrence could be detected later. A prospective trial comparing injection schemes to protocolled injections using objective scar assessment could provide the necessary clarification.

### Combined regimens with and without cryotherapy

In this cohort, a novel approach to combined cryotherapy regimens has been employed since 2018. The technique consisted of weekly scheduled intralesional corticosteroid injections followed by surgical excision of keloid ± four to six weeks after the last injection. This was finally followed by the application of cryotherapy to the new scar tissue approximately two weeks after suture removal. Intralesional cryotherapy was initially administered through Cryoshape needles, and from April 2019 onwards, through external administration – owing to the unavailability of Cryoshape needles in the Netherlands.

One initial aim of this study was to assess this novel scheme. However, we failed to include sufficient cases for a proper analysis. Therefore, we decided to compare combined regimens with cryotherapy to combinations without cryotherapy. Our results exhibited superior efficacy of combined cryotherapy regimens compared to monotherapy, but this was not statistically significant. Cryotherapy has demonstrated better efficacy as a part of combined regimens than when applied as a standalone treatment. As cryotherapy induces differentiation of abnormal keloid tissue to normal phenotype,^7^^,^^12^ its application is best suitable as an adjunctive therapy or for the treatment of keloids in their early stages of development.^12^^,^^13^

### Strengths and Limitations

This study was conducted on a relatively large population with 204 keloid scars, which provided large groups for primary comparison of combined to monotherapies. Average follow-up at 430 days (61 weeks) and frequent follow-up visits provided an overview of recurrence and treatment response. Furthermore, this study introduced unique concepts of corticosteroid injection schemes and combined regimen schemes for injections, surgical excision, and cryotherapy for treating keloids.

The main limitations of this study are its retrospective nature and the subjective outcome measures to scar assessment. Selection bias was applied as there was no randomization to the different treatment groups. It was also not possible to inquire about the occurrence of side effects or adverse reactions from the charts as their occurrence was not documented, nor was it specifically stated when they did not occur. Therefore, it is not possible to conclude whether a patient experienced adverse effects. While most cases did have frequent follow-up visits in which keloid was mentioned multiple times, explicit differentiation from hypertrophic scars was lacking and we were uncertain whether cases of hypertrophic scars incorrectly diagnosed as keloids were included in this cohort. Future prospective research should be carried out to address these issues by applying objective measurements and validated scar assessment scales, and randomizing patients to treatment groups which will render more reliable results.

## Declaration of Competing Interest

The authors declare that they have no conflict of interest.
